# Vestibular Test Results in Patients With Horizontal Canal Benign Paroxysmal Positional Vertigo

**DOI:** 10.7759/cureus.21460

**Published:** 2022-01-20

**Authors:** Eric K Kim, Lauren Pasquesi, Kristen K Steenerson, Jorge Otero-Millan, Jeffrey D Sharon

**Affiliations:** 1 Otolaryngology - Head and Neck Surgery, University of California San Francisco School of Medicine, San Francisco, USA; 2 Otolaryngology - Head and Neck Surgery, University of California San Francisco, San Francisco, USA; 3 Otolaryngology - Head and Neck Surgery, Stanford University, Stanford, USA; 4 Neurology, Stanford University, Stanford, USA; 5 School of Optometry, University of California Berkeley, Berkeley, USA; 6 Neurology, Johns Hopkins University, Baltimore, USA

**Keywords:** utricle, horizontal semicircular canal, vertigo, vestibular, bppv

## Abstract

Introduction

While the mechanism of posterior canal benign paroxysmal positional vertigo (BPPV) is widely accepted as canalolithiasis, the pathophysiology of horizontal canal BPPV remains controversial. We seek to analyze vestibular test results of patients with horizontal canal BPPV with ageotropic nystagmus (AHC) and geotropic nystagmus (GHC) in comparison to patients with posterior canal BPPV (PC) to better understand its pathophysiology.

Methods

In a retrospective chart review of adults with BPPV at a tertiary referral balance center, we reviewed the clinical characteristics and compared videonystagmography, caloric, rotary chair, subjective visual vertical (SVV)/ subjective visual horizontal (SVH), and vestibular evoked myogenic potential (VEMP) results between groups.

Results

We included 11 AHC and seven GHC patients and randomly selected 20 PC patients as the comparison group. All groups had a high rate of migraine and low rates of diabetes and head trauma, but no difference between groups. Ipsilateral caloric weakness was more prevalent in the GHC group compared to the PC group (p=0.02). One of two AHC patients and both GHC patients who had SVV/SVH testing had abnormal findings. The only AHC patient who had ocular VEMP testing had abnormal results. Additionally, we observed a significant downbeating component to nystagmus (4 deg/sec or greater) exclusively in the AHC group (5/10 patients, p=0.001).

Conclusions

Patients with AHC and GHC have unique vestibular testing results. In particular, only AHC patients showed a downbeating component to their nystagmus, which may suggest utricular dysfunction in the pathophysiology of AHC.

## Introduction

Benign paroxysmal positional vertigo (BPPV) is the most common peripheral vestibular disorder, causing attacks of vertigo with positional changes. The estimated lifetime prevalence is 2.4%, and BPPV has been associated with a significant impact on quality of life [1,2]. The most common form of BPPV involves the posterior semicircular canals, which account for 85% of cases [1]. The prevalence of horizontal canal BPPV (HC-BPPV) may have been underestimated due to a higher spontaneous resolution rate [3]. BPPV involving the horizontal canal has two variants: one with geotropic nystagmus (beating toward the ground) and another with ageotropic nystagmus (beating away from the ground).

Two different processes are thought to mediate the characteristic nystagmus seen in the two subtypes of HC-BPPV. The pathogenesis of the geotropic nystagmus variant is thought to be due to canalolithiasis: displacement of otoconia (calcium carbonate crystals) into the fluid of the membranous labyrinth of the horizontal canal [4]. A head turn toward the impaired side during the supine roll test will create an ampullopetal (excitatory) endolymph flow due to downward displacement of otoconia towards the ampulla in the lower ear. This causes a stronger nystagmus than a turn away from the affected side, which creates an ampullofugal (inhibitory) stimulus due to otoconia falling away from the cupula, this time in the upper ear. For ageotropic nystagmus, it has been theorized that otoconia adhere to the cupula, which is termed cupulolithiasis. When the patient turns toward the impaired side, there is an ampullofugal stimulus due to the heavy cupula falling away from the vestibule in the lower ear, which results in an ageotropic nystagmus. A head turn to the healthy side will induce an ampullopetal stimulus due to the heavy cupula falling towards the vestibule in the upper ear, which in turn causes an even stronger ageotropic nystagmus [5].

One case series of patients with BPPV identified that 25% of the subjects had canal paresis at diagnosis [6], whereas another study found that 20.4% of BPPV patients exhibited caloric hypo-excitability [7]. However, there is limited literature that characterizes other vestibular testing results of patients with HC-BPPV. The objective of this study is to identify patterns of clinical factors and vestibular test results of both geotropic and ageotropic HC-BPPV to gain a deeper understanding of the pathogenesis of horizontal canal BPPV. This article was previously presented as a poster at the 2021 COSM American Otological Society conference on April 7-11, 2021.

## Materials and methods

Subjects

We conducted a retrospective chart review of patients seen at the University of California, San Francisco (UCSF) Balance and Falls Center from 01/2015 to 06/2020 to identify patients with horizontal canal BPPV. This study was approved by the UCSF IRB (IRB 18-25365). Inclusion criteria included patients who had 1) diagnosis of horizontal canal BPPV, 2) vestibular testing results (at minimum videonystagmography with caloric testing), and 3) were aged ≥ 18 years. Using the same inclusion criteria, 20 posterior canal BPPV patients with vestibular test results were selected using a random number generator as a comparison group. Patients with central causes of vertigo or central nervous system (CNS) disorders were excluded.

Diagnosis

Diagnosis of BPPV was made in consultation with Barany Society guidelines [8] by audiologists or otolaryngology providers. For HC-BPPV, the following criteria were used to characterize its nystagmus, which was reported in the primary gaze position: 1) positional horizontal geotropic nystagmus with fast phase toward the ground when turning to either side during supine roll testing; 2) ageotropic nystagmus with horizontal nystagmus with fast phase directed away from the ground with head turning during supine roll testing. For the geotropic nystagmus variant, the side (right ear down or left ear down) with stronger symptoms and nystagmus was assumed to be caused by the lower ear; for the ageotropic nystagmus variant, the opposite was true (the upper ear on the side with stronger symptoms and nystagmus was assumed to be the cause) [9]. HC-BPPV was differentiated from central positional vertigo by confirming each patient’s clinical improvement after treatment. Posterior canal BPPV was diagnosed when a positional geotropic, torsional upbeating nystagmus was elicited with the Dix-Hallpike maneuver.

Clinical history and vestibular tests

For each patient, we reviewed the clinical history questionnaire and vestibular test results. As relevant, past medical history and patient history of migraine, diabetes mellitus, and head injury were recorded. Further, we collected the patient’s Dizziness Handicap Index (DHI), self-reported duration of BPPV symptoms, and whether the symptoms began with a severe episode. The tests of interest were caloric, rotary chair, subjective visual vertical (SVV), subjective visual horizontal (SVH), and vestibular evoked myogenic potential (VEMP) tests. Providers independently decided whether to order SVV and SVH for a patient, which were performed prior to any positional testing. VEMP was performed only when specifically ordered by the referring provider. Caloric weakness was defined as 20% or greater asymmetry as calculated by Jongkees formula. Rotary chair testing was determined to be abnormal if any of the following parameters was outside the normal ranges: sinusoidal (gain, phase, symmetry, visual enhancement, suppression) and trapezoidal (gain and time constant). Cutoff values for normal SVV and SVH were 2.5 degrees and 3 degrees, respectively. The presence of significant positional downbeating component to nystagmus (4 deg/sec or greater) with supine roll tests was also noted. Recordings of the downbeating nystagmus were reviewed by an experienced audiologist, who confirmed that there were no torsional components. Because not all patients had complete datasets, only the available data was included for analysis.

Data and statistics

Normal distribution for the DHI was confirmed with quantile-quantile plots. Descriptive statistics (means, range, and standard deviations) were used to characterize quantitative variables including age and DHI. Fisher’s exact test and analysis of variance (ANOVA) test followed by pairwise comparisons in Stata (Version 16.1) (StataCorp LLC, College Station, Texas, USA) were used to compare sex, self-reported duration of symptoms, nature of symptom onset, and histories of migraine, head trauma, and diabetes mellitus. P-value under 0.05 was considered statistically significant.

## Results

Between January 2015 and June 2020, we identified 560 patients diagnosed with BPPV seen at the Balance and Falls Center. In this cohort, 28 patients had HC-BPPV with an overall prevalence of 5%. Eighteen patients with HC-BPPV met the inclusion criteria. Eleven patients had horizontal canal BPPV with ageotropic nystagmus (AHC), and seven patients had horizontal canal BPPV with geotropic nystagmus (GHC). As a comparison group, 20 posterior canal BPPV (PC) patients were randomly selected.

Clinical history and risk factors

The mean age of the study population was 62 ± 14 years (range: 28-84), and 63% of patients were female. There were no statistical differences in age and sex between the groups (p>0.05). Baseline characteristics of each group are described in Table 1.

**Table 1:** Baseline characteristics of the study cohort

	Horizontal Canal BPPV with Ageotropic Nystagmus (AHC) (n=11)	Horizontal Canal BPPV with Geotropic Nystagmus (GHC) (n=7)	Posterior Canal BPPV (PC) (n=20)	p-value
Mean age ± SD, yr	67 ± 10	55 ± 15	61 ± 15	0.256
Female sex – no. (%)	6/11 (54%)	3/7 (43%)	15/20 (75%)	0.247
Dizziness Handicap Index (DHI) ± SD	26 ± 11	41 ± 16	41 ± 25	0.143
Self-reported duration of symptoms				
Seconds – no. (%)	3 (27%)	2 (29%)	9 (45%)	0.641
Minutes – no. (%)	3 (27%)	4 (57%)	6 (30%)	
Hours – no. (%)	2 (18%)	0 (0%)	1 (5%)	
Days – no. (%)	2 (18%)	1 (14%)	1 (5%)	
Constant – no. (%)	1 (9%)	0 (0%)	3 (15%)	
Began with a severe episode – no. (%)	5/9 (56%)	4/7 (57%)	4/8 (50%)	1.000
History of migraine – no. (%)	4/11 (36%)	5/7 (71%)	10/20 (50%)	0.393
History of head trauma – no. (%)	1/11 (9%)	2/7 (29%)	3/20 (15%)	0.609
History of diabetes mellitus – no. (%)	0/11 (0%)	1/7 (14%)	3/20 (15%)	0.456

The DHI for the study population was 36 ± 21 (range: 0-90), which corresponds to moderate functional impairment [10]. When analyzing the AHC and GHC groups together, the DHI was 32 ± 15 and was similar to the mean DHI of the PC group (p=0.193). Individually, DHI was similar between the three groups (p=0.14). The self-reported duration of vertigo was widely variable from seconds to days within each group, but there were no differences between groups (p=0.64). BPPV began with a severe episode in 56% of AHC, 57% of GHC, and 50% of PC patients (p=1.00).

Overall, a history of migraine was present for 50% of all patients. Individually, all three groups showed a high rate of migraine but did not show statistical difference (36% AHC; 71% GHC; 50% PC, p=0.35). A history of head injury was not common (9% AHC; 29% GHC; 15% PC, p=0.54). Diabetes mellitus was also not prevalent among patients in all groups (0% AHC; 14% GHC; 15% PC, p=0.46).

Videonystagmography

**Figure 1:**
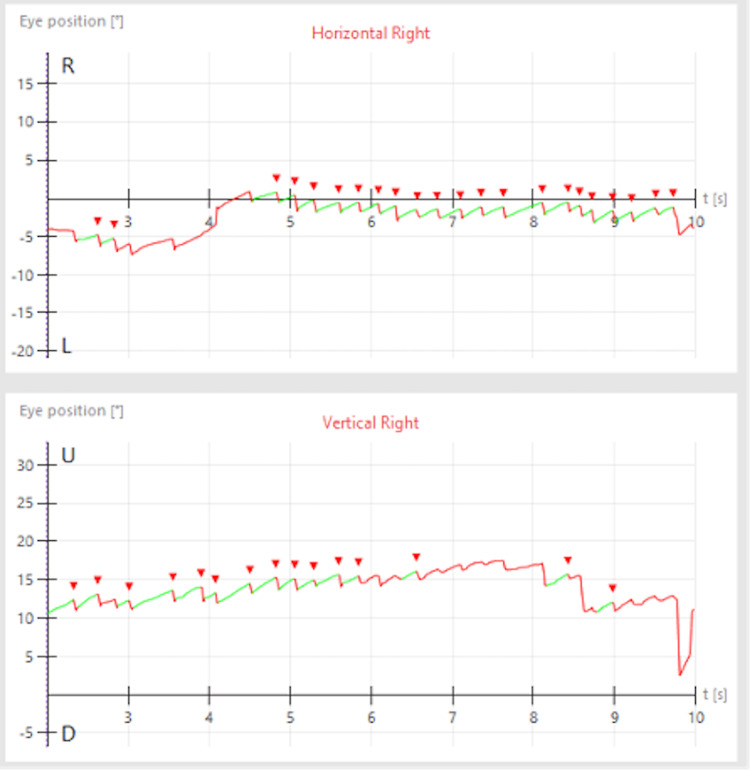
A sample videonystagmography tracing of a patient with ageotropic nystagmus elicited in the body right position.

Ipsilateral caloric weakness was significantly more prevalent in the GHC group compared to the PC group (p=0.02). It was observed in 43% of the AHC and 71% of the GHC groups, compared to the 20% in the PC group. Five of ten AHC patients had a downbeating component in their nystagmus greater than or equal to 4 deg/sec triggered by the maneuver, while no individuals in the other two groups showed this finding (p=0.001). All vestibular test results are summarized in Table 2. A sample videonystagmography tracing of an AHC patient with the downbeat nystagmus is shown in Figure 1.

**Table 2:** Vestibular testing results of each study group SVV: Subjective Visual Vertical; SVH: Subjective Visual Horizontal; N/A: Not applicable. †: Excluded one patient because the audiology report stated that detection of downbeating nystagmus was due to provocation of BPPV. *: Statistically significant.

	Horizontal Canal BPPV with Ageotropic Nystagmus (AHC) (n=11)	Horizontal Canal BPPV with Geotropic Nystagmus (GHC) (n=7)	Posterior Canal BPPV (PC) (n=20)	p-value
Ipsilateral caloric weakness – no. (%)	4/7 (43%)	5/7 (71%)	4/20 (20%)	0.038*
Abnormal rotary chair – no. (%)	5/9 (56%)	5/7 (71%)	9/18 (50%)	0.668
Abnormal SVV/SVH	1/2 (50%)	2/2 (100%)	0/2 (0%)	0.135
Absent VEMP	1/1 (100%)	N/A	0/1 (0%)	N/A
Significant downbeating component of nystagmus – no. (%)	5/10 (50%)	0/7 (0%)	0/19 (0%)^†^	0.001*

Rotary chair

All three groups had high rates of abnormal rotary chair testing results, although there was no overall statistical difference (56% AHC, 71% GHC, 50% PC, p=0.63). No specific patterns in sinusoidal and trapezoidal testing abnormalities emerged within or between groups.

Subjective visual vertical/subjective visual horizontal

A total of six patients (two patients in each respective group) underwent SVV and SVH testing. In the AHC group, one of the two individuals had abnormal findings. Patient A had an average deviation of -8.21 degrees of counterclockwise skew on SVV and average deviation of -10.13 degrees of counterclockwise skew on SVH. In the GHC group, both patients had abnormal results. Patient B had an average deviation of -4.5 degrees of counterclockwise skew on SVV and an average deviation of -3.4 degrees of counterclockwise skew on SVH. Patient C had an average deviation of 3.437 degrees of clockwise skew on the SVH. Two patients with PC had normal results. Cases with abnormal SVV/SVH results are further shown in Table 3.

**Table 3:** List of patients with abnormal Subjective Visual Vertical, Subjective Visual Horizontal, or VEMP testing results. SVV: Subjective Visual Vertical; SVH: Subjective Visual Horizontal; AHC: Horizontal canal BPPV with ageotropic nystagmus; GHC: Horizontal canal BPPV with geotropic nystagmus; VEMP: Vestibular evoked myogenic potential; N/A: Not applicable; BPPV: Benign paroxysmal positional vertigo.

Patient	Age	Sex	Diagnosis	SVV	SVH	Ocular VEMP results
A	69	F	Left AHC	Average deviation of -8.21 degrees of counterclockwise skew	Average deviation of -10.13 degrees of counterclockwise skew	N/A
B	56	M	Right GHC	Average deviation of -4.5 degrees of counterclockwise skew	Average -3.4 degrees of counterclockwise skew	N/A
C	71	M	Right GHC	Normal	Average deviation of 3.437 degrees of clockwise skew	N/A
D	74	F	Right AHC	Normal	Normal	Absent on the right

Vestibular evoked myogenic potential (VEMP)

One patient in the AHC group and another patient in the PC group completed VEMP testing. As seen in Table 3, Patient D (woman with right AHC and hearing sensitivity within normal limits) had absent ipsilateral ocular VEMP (oVEMP) and present contralateral ocular VEMP. The patient with PC had normal findings.

## Discussion

At our dizziness clinic, approximately 5% of the BPPV cases involved the horizontal canal, and the incidences of AHC and GHC were 39% and 61% respectively, which align with the findings of other studies examining HC-BPPV [5,11]. HC-BPPV has been shown to have a higher rate of spontaneous resolution than posterior canal BPPV (PC-BPPV), which may explain its relatively lower frequency [12].

HC-BPPV has been associated with a greater severity of symptoms and therefore an earlier presentation to the clinic for evaluation and treatment [11]. One observational study suggested that HC-BPPV was associated with a longer duration of symptoms and patient-perceived disability as quantified by the DHI [13]. However, our results did not demonstrate a difference in DHI or duration of symptoms between the 18 HC-BPPV patients (combined groups of AHC and GHC) and the 20 PC patients. When we separate out the two subtypes of HC-BPPV, we still did not observe a difference in the mean DHI scores between the three groups. Although the DHIs of the three groups in our study were consistent with the literature, our findings suggest that no one type of BPPV is reliably correlated with a more severe dizziness-related quality of life [10].

In our cohort, 67% of HC-BPPV and 75% of PC-BPPV patients reported that their symptoms lasted seconds to minutes. While this transient time course of symptoms matches the classic description of BPPV symptoms as transient in the majority of patients, 22% of HC-BPPV and 20% of PC-BPPV patients experienced vertigo for days or constantly. This wide variability in presentation has also been recognized in the Barany Society guidelines [8] and stresses the importance of recognizing that BPPV, regardless of the canal involved, presents in various ways.

In our review of patients’ past medical history, we focused on known risk factors for BPPV, such as migraine, diabetes, and a personal history of head injury [14]. All three groups had a low rate of diabetes and head injury, with no statistically significant differences. Notably, all groups had a high prevalence of migraine, which is consistent with large epidemiological studies demonstrating that migraine is associated with an increased risk for BPPV [15]. Patients with migraine are also at an increased risk of developing recurrent BPPV symptoms [16]. To explain the comorbidity of migraine and BPPV, Ishiyama et al. have proposed that vasospasm in migraine causes repeated damage to the vasculature of the inner ear, which predisposes to the displacement of otoconia [17]. While the common pathophysiology remains unclear, our data further supports the link between the two conditions. We did not identify any specific risk factors that seem to predispose individuals to develop a certain type of BPPV. This study was not designed to assess other known risk factors, such as osteoporosis/osteopenia, vitamin D deficiency, or other inner ear pathologies.

The mechanism of HC-BPPV with ageotropic nystagmus remains unclear. Cupulolithiasis was first described in 1969, when Schuknecht found debris attached to the posterior surface of the cupula in two patients with BPPV and suggested that the adherent particles make the cupula gravity-dependent [18]. When the impaired ear is down, the “heavy” cupula would move away from the utricle and decrease firing of the ampullary nerve. Conversely, when the patient turns toward the unaffected side, the cupula would deviate toward the utricle and produce a nystagmus toward the uppermost ear. In contrast, it seems fairly clear at this point that canalolithiasis accounts for cases of posterior canal and most horizontal canal geotropic nystagmus [19].

Nuti et al. have proposed canalolithiasis as an alternative theory for AHC; with an initial location closer to the ampulla than in the geotropic nystagmus variant, the otoconia particles would move in the opposite direction from the geotropic variant and produce ageotropic nystagmus [20]. These theories, however, have limitations and may not explain the downbeating component nystagmus that we observed, as the horizontal canal should produce a purely horizontal nystagmus when eyes are aligned with the plane of the canals, as per Ewald’s first law.

Utricular dysfunction may explain our findings and play a role in the pathomechanism of AHC. It is normally assumed that loss of otolith function results in the triad of the ocular tilt response [21]. While controversial, Curthoys et al. have argued that a sudden loss of otolith function can present with nystagmus in a case report and small case series [22,23]. Utricular damage alone, however, may not explain the phenomenon of AHC. The brain integrates information from both the semicircular canals and the otolith to resolve the tilt-translation ambiguity. Angelaki et al. demonstrated that animals whose semicircular canals were inactivated could not discriminate roll tilt and translation, resulting in similar horizontal eye movements [24]. Extending that logic, we wonder if unmasked utricular asymmetry during positional testing in patients with already injured semicircular canals results in ageotropic horizontal nystagmus, rather than cupulolithiasis. This would help account for the high rate of unilateral vestibular loss seen in our cohort, difficulties with verticality perception, and may explain the downbeat component of the nystagmus that was observed. Using cats, Goto et al. showed that stimulation of the utricular nerve evoked horizontal eye movements to the ipsilateral side as well as oblique movements to the upper contralateral side [25]. Several other studies have also characterized the human translational vestibulo-ocular reflex, which is anatomically mediated by connections between the utricle and the abducens nucleus [26,27]. The oblique eye movements observed in Goto et al.’s study may be closely related to the downbeating nystagmus component we observed with the AHC patients in this present study. Although we have VEMP data for just one patient with AHC, oVEMP was shown to be absent in this patient, which further suggests utricular involvement in AHC. We therefore hypothesize that the ageotropic horizontal nystagmus may also be related to utricular afferent asymmetries in the context of damaged semicircular canals triggering nystagmus. This may also help explain why ageotropic horizontal canal nystagmus is resistant to treatment.

Alternatively, the horizontal nystagmus with the downbeat component may also be explained by a combined superior and horizontal canal BPPV. In Lopez-Escamez et al.’s cohort study, 10% of the subjects showed positional horizontal nystagmus with a vertical side-changing component, which they attributed to multicanal BPPV [28]. With the involvement of the superior canal, however, we would expect a torsional component to the downbeat nystagmus as per the Barany consensus statement. We confirmed its absence in the AHC patients, which makes multicanal BPPV a less likely explanation [29,30].

There were several notable limitations in this study. First, we had a small sample size of patients with HC-BPPV. Even in a large cohort of 560 patients over a 5.5-year data collection period, we identified only 28 with the diagnosis, which matches the small percentages of HC-BPPV patients in other studies. The study might have been underpowered to detect differences in risk factors and testing parameters between the three groups. The DHI data also did not perfectly fit a normal distribution, most likely because of the smaller sample size. Moreover, not all patients presenting to the clinic for evaluation received the same battery of vestibular testing results. Because not everyone with findings concerning for AHC received VEMP or SVV testing, it was challenging to establish definitive patterns of VEMP and SVV in this population. Another concern is the generalizability of our results, which pertain to only tertiary referral settings.

## Conclusions

While there were no statistically significant differences in clinical characteristics or medical histories between AHC, GHC, and PC groups, we observed a significant component of positional downbeating nystagmus was observed exclusively in AHC patients. In line with previous studies that show that utricular stimulation causes diagonal nystagmus, the non-torsional downbeat nystagmus seen in AHC patients suggests that the utricle may be involved in the pathophysiology of AHC, rather than the horizontal canal cupula. Multicanal BPPV is also a possible explanation, although less likely considering the lack of a rotary component to the downbeat.

## References

[1] Hornibrook J (2011). Benign paroxysmal positional vertigo (BPPV): history, pathophysiology, office treatment and future directions. Int J Otolaryngol.

[2] Gámiz MJ, Lopez-Escamez JA (2004). Health-related quality of life in patients over sixty years old with benign paroxysmal positional vertigo. Gerontology.

[3] Kim JS, Zee DS (2014). Benign paroxysmal positional vertigo. N Engl J Med.

[4] Baloh RW, Jacobson K, Honrubia V (1993). Horizontal semicircular canal variant of benign positional vertigo. Neurology.

[5] Parnes LS, Agrawal SK, Atlas J (2003). Diagnosis and management of benign paroxysmal positional vertigo (BPPV). CMAJ.

[6] Molina MI, López-Escámez JA, Zapata C, Vergara L (2007). Monitoring of caloric response and outcome in patients with benign paroxysmal positional vertigo. Otol Neurotol.

[7] Yetişer S, İnce D (2017). Caloric analysis of patients with benign paroxysmal positional vertigo. J Int Adv Otol.

[8] von Brevern M, Bertholon P, Brandt T, Fife T, Imai T, Nuti D, Newman-Toker D (2015). Benign paroxysmal positional vertigo: diagnostic criteria. J Vestib Res.

[9] Han BI, Oh HJ, Kim JS (2006). Nystagmus while recumbent in horizontal canal benign paroxysmal positional vertigo. Neurology.

[10] Whitney SL, Marchetti GF, Morris LO (2005). Usefulness of the dizziness handicap inventory in the screening for benign paroxysmal positional vertigo. Otol Neurotol.

[11] Chung KW, Park KN, Ko MH (2009). Incidence of horizontal canal benign paroxysmal positional vertigo as a function of the duration of symptoms. Otol Neurotol.

[12] Imai T, Ito M, Takeda N, Uno A, Matsunaga T, Sekine K, Kubo T (2005). Natural course of the remission of vertigo in patients with benign paroxysmal positional vertigo. Neurology.

[13] Martens C, Goplen FK, Aasen T, Nordfalk KF, Nordahl SH (2019). Dizziness handicap and clinical characteristics of posterior and lateral canal BPPV. Eur Arch Otorhinolaryngol.

[14] Chen J, Zhang S, Cui K, Liu C (2021). Risk factors for benign paroxysmal positional vertigo recurrence: a systematic review and meta-analysis. J Neurol.

[15] Kim SK, Hong SM, Park IS, Choi HG (2019). Association between migraine and benign paroxysmal positional vertigo among adults in South Korea. JAMA Otolaryngol Head Neck Surg.

[16] Bruss D, Abouzari M, Sarna B, Goshtasbi K, Lee A, Birkenbeuel J, Djalilian HR (2021). Migraine features in patients with recurrent benign paroxysmal positional vertigo. Otol Neurotol.

[17] Ishiyama A, Jacobson KM, Baloh RW (2000). Migraine and benign positional vertigo. Ann Otol Rhinol Laryngol.

[18] Schuknecht HF (1969). Cupulolithiasis. Arch Otolaryngol.

[19] Kao WT, Parnes LS, Chole RA (2017). Otoconia and otolithic membrane fragments within the posterior semicircular canal in benign paroxysmal positional vertigo. Laryngoscope.

[20] Nuti D, Mandalà M, Salerni L (2009). Lateral canal paroxysmal positional vertigo revisited. Ann N Y Acad Sci.

[21] Wolfe GI, Taylor CL, Flamm ES, Gray LG, Raps EC, Galetta SL (1993). Ocular tilt reaction resulting from vestibuloacoustic nerve surgery. Neurosurgery.

[22] Curthoys IS, Burgess AM, Manzari L (2020). The evidence for selective loss of otolithic function. Semin Neurol.

[23] Manzari L, Burgess AM, Curthoys IS (2012). Does unilateral utricular dysfunction cause horizontal spontaneous nystagmus?. Eur Arch Otorhinolaryngol.

[24] Angelaki DE, McHenry MQ, Dickman JD, Newlands SD, Hess BJ (1999). Computation of inertial motion: neural strategies to resolve ambiguous otolith information. J Neurosci.

[25] Goto F, Meng H, Bai R, Sato H, Imagawa M, Sasaki M, Uchino Y (2003). Eye movements evoked by the selective stimulation of the utricular nerve in cats. Auris Nasus Larynx.

[26] Bronstein AM, Gresty MA (1988). Short latency compensatory eye movement responses to transient linear head acceleration: a specific function of the otolith-ocular reflex. Exp Brain Res.

[27] Ramat S, Zee DS (2003). Ocular motor responses to abrupt interaural head translation in normal humans. J Neurophysiol.

[28] Lopez-Escamez JA, Molina MI, Gamiz M, Fernandez-Perez AJ, Gomez M, Palma MJ, Zapata C (2005). Multiple positional nystagmus suggests multiple canal involvement in benign paroxysmal vertigo. Acta Otolaryngol.

[29] Herdman SJ (1997). Advances in the treatment of vestibular disorders. Phys Ther.

[30] Eggers SD, Bisdorff A, von Brevern M (2019). Classification of vestibular signs and examination techniques: nystagmus and nystagmus-like movements. J Vestib Res.

